# Efficacy of Alpinumisoflavone Isolated from *Maclura tricuspidata* Fruit in Tumor Necrosis Factor-α-Induced Damage of Human Dermal Fibroblasts

**DOI:** 10.3390/antiox10040514

**Published:** 2021-03-25

**Authors:** Sullim Lee, Giang Do Hoang, Daeyoung Kim, Ho Sueb Song, Sungyoul Choi, Dongho Lee, Ki Sung Kang

**Affiliations:** 1 Department of Life Science, College of Bio-Nano Technology, Gachon University, Seongnam 13120, Korea; sullimlee@gachon.ac.kr (S.L.); davekim@gachon.ac.kr (D.K.); 2 Department of Plant Biotechnology, College of Life Sciences and Biotechnology, Korea University, Seoul 02841, Korea; giangdh.91@gmail.com; 3 College of Korean Medicine, Gachon University, Seongnam 13120, Korea; hssong70@gachon.ac.kr (H.S.S.); pc1075@gachon.ac.kr (S.C.)

**Keywords:** skin aging, ROS, TNF-α, human dermal fibroblasts, *Maclura tricuspidata* fruit, alpinumisoflavone

## Abstract

The skin is an important organ in the human body that protects the body from environmentally hazardous substances. Reactive oxygen species (ROS) cause inflammatory reactions and degradation of the extracellular matrix leading to skin aging and various cutaneous lesions. This study evaluated the potential of isoflavones isolated from *Maclura tricuspidata* fruit to prevent TNF-α-induced skin inflammation in normal human dermal fibroblasts (HDFs). It focused on alpinumisoflavone (AIF) that suppressed the accumulation of ROS and nitric oxide (NO) in tumor necrosis factor-alpha (TNF-α)-treated HDFs. AIF inhibited the TNF-α-induced increase in matrix metalloproteinase-1, decreased procollagen I α1, and suppressed pro-inflammatory mediators and pro-inflammatory cytokines, including NO synthase, cyclooxygenase-2, interleukin (IL)-1β, IL-6, and IL-8 that trigger inflammatory responses. AIF inhibited nuclear factor-κB and activating protein 1 mitogen-activated protein kinases that were increased by TNF-α stimulation. These results suggest that AIF may protect skin from aging and various cutaneous lesions.

## 1. Introduction

Skin is the primary protector of the human ectoderm system, is in direct contact with potentially harmful factors, and performs three major functions: skin sensation, control, and protection [1]. Intrinsic aging involves damage that occurs over time due to a decrease in skin cell activity caused by reactive oxygen species (ROS) produced during the skin cells’ metabolism [2]. Extrinsic aging is induced by exposure to external environmental hazards, such as pollution, chemicals, smoking, and ultraviolet (UV) light [3]. Skin damage and aging result from its direct exposure to the external environment [1]. UV irradiation is considered to be an important factor in inducing various skin conditions, such as skin aging and inflammatory skin diseases [4,5]. It causes extensive inflammatory damage to the skin from the epidermis to the dermis [6] due to ROS production, leading to cumulative skin damage, such as sunburn, photoaging, and skin pigmentation [7].

The production of ROS has been reported to cause oxidative damage and affect biological functions, such as cell membrane destruction, DNA denaturation, inflammatory response, and immunodeficiency [8,9,10]. ROS are generated by oxidative phosphorylation in mitochondria. Detrimental foreign material can induce the accumulation of ROS [11]. Excess ROS in the skin leads to wrinkles formation through chain crossing errors and cleavage of fibrous proteins, such as elastic fibers and collagen that comprise the extracellular matrix (ECM) of the skin [12]. Collagenases like matrix metalloproteinase-1 (MMP-1) inhibit collagen synthesis [13]. The main cause of extrinsic aging is an ROS-mediated secondary reaction that occurs when UV radiation is absorbed by the skin [14]. Antioxidants that inhibit the generation of ROS and MMP-1 may protect the skin from aging.

Exposure of the skin to UV radiation produces cyclooxygenase-2 (COX-2), inducible nitric oxide synthase (iNOS), tumor necrosis factor-alpha (TNF-α), interleukin (IL)-1β, IL-6, and IL-8 [15,16,17] that can directly or indirectly induce pro-inflammatory mediators. In particular, the inflammatory-response damage of skin fibroblasts accelerates the process of photoaging [15,18]. TNF-α is an important oxidative stress and inflammation mediator in the skin. UVB-induced TNF-α secretion stimulates keratinocytes and fibroblasts to express cell adhesion molecules, recruits immune cells to create collagenase, and causes skin aging and damage. Collagen degradation can result in unwanted changes in appearance, including deep wrinkles, sagging, and atrophied skin [19]. Chronic inflammation induced by UV irradiation can also increase the risk of various skin diseases. Numerous studies on reducing skin wrinkles have focused on the inhibitory activity of collagenase [20,21,22].

*Maclura tricuspidata* Carr. (syn. *Cudrania tricuspidata*) is a deciduous tree belonging to the family Moraceae; it has been found in various medicinal and nutritional applications in East Asia. Prenylated isoflavones and xanthones are considered the major constituents of *M. tricuspidata* and have been shown to have various biological activities, including anti-inflammatory, antioxidant, antiatherosclerotic, hepatoprotective, and neuroprotective effects.

Our preliminary experiment showed that compounds isolated from *Maclura tricuspidatan* possessed anti-oxidative and 2,2-diphenyl-1-picrylhydrazyl (DPPH)-scavenging properties. Previous studies reported that the antioxidant activity of isoflavones may regulate oxidative damage within cells, implying that isoflavones may prevent oxidative stress-related damage in the skin ECM [23,24]. We used human dermal fibroblasts (HDFs) to investigate the anti-aging effects of isoflavones isolated from *M. tricuspidata* fruit. In the present study, we describe the antioxidant and anti-aging effects of isoflavones on TNF-α-stimulated HDFs and identify the mechanisms of active compounds.

## 2. Materials and Methods

### 2.1. Plant Material and Isolation of Compounds from Maclura tricuspidata Fruit

A voucher specimen (accession number KH1-4-090814) was deposited at the Department of Plant Biotechnology, Korea University, Seoul, Korea. *M. tricuspidata* fruit extract (MTF), alpinumisoflavone (AIF), and 4′-*O*-methyl alpinumisoflavone (MAIF) were prepared from the fruits of *M. tricuspidata* as previously described [25].

### 2.2. DPPH Free Radical Scavenging

The indicated concentrations of samples were dissolved in dimethyl sulfoxide (DMSO) and mixed with 100 µL of DPPH solution in a 96-well plate. After reacting for 30 min in dark conditions, the reaction results were read on a microplate reader (SPARK 10M; Tecan, Männedorf, Switzerland) at 517 nm. Data are presented as means ± standard error of mean (SEM) of three independent experiments performed in triplicate.

### 2.3. HDFs Cultures

Human dermal fibroblast HDFs were obtained from PromoCell GmbH (Sickingenstr, Heidelberg, Germany). Cells were maintained at 37 °C in a humidified, 5% CO_2_ atmosphere in Dulbecco’s modified Eagle’s medium (Corning, Manassas, VA, USA) supplemented with 10% fetal bovine serum (Atlas, Fort Collins, CO, USA) and penicillin/streptomycin (Gibco, Grand Island, NY, USA) antibiotics.

### 2.4. Scavenging of Intracellular ROS in TNF-α-Treated HDF

Experiments were conducted as previously described [26]. Briefly, HDFs were plated at a density of 1 × 10^4^ cells/well in 96-well plates, allowed to adhere overnight, and then starved for 24 h under serum-free conditions. HDFs were challenged with 20 ng/mL TNF-α (PeproTech, Rocky Hill, NJ, USA) in the presence or absence of MTF (12.5 and 25 µg/mL), AIF (25 and 50 µM), MAIF (25 and 50 µM), and 10 µM DCFDA (Sigma-Aldrich, St. Louis, MO, USA). The reaction results were read on a microplate reader (SPARK 10M; Tecan, Männedorf, Switzerland) at an excitation and emission spectra of 495 nm and 529 nm. Data are presented as means ± SEM of three independent experiments performed in triplicate.

### 2.5. Scavenging of NO in TNF-α-Treated HDF

Experiments were conducted as previously described [27]. Briefly, HDFs were plated at a density of 1 × 10^4^ cells/well in 96-well plates, allowed to adhere overnight, and then starved for 24 h under serum-free conditions. HDFs were challenged with 20 ng/mL TNF-α in the presence or absence of MTF (12.5 and 25 µg/mL), AIF (25 and 50 µM), and MAIF (25 and 50 µM) for 24 h. The nitrite content of the supernatant was quantified using the Griess assay. The supernatant was incubated with 1% sulfanilamide, 0.1% *N*-(1-naphthyl)-ethylenediamine, and 5% phosphoric acid at room temperature for 10 min. Thereafter, nitrite content was read on a microplate reader using 540 nm wavelength. NO production in each sample was then calculated using a standard sodium nitrite (NaNO_2_) curve.

### 2.6. Detection of Proteins Secretion in TNF-α-Treated HDF

Experiments were conducted as previously described [28]. Briefly, HDFs were plated at a density of 4 × 10^4^ cells/well in 48-well plates, allowed to adhere overnight, and then starved for 24 h under serum-free conditions. HDFs were challenged with 20 ng/mL TNF-α in the presence or absence of MTF (12.5 and 25 µg/mL), AIF (25 and 50 µM), and MAIF (25 and 50 µM) for 12 h (for IL-1β, IL-6, and IL-8) and 24 h (for MMP-1 and COLIA1). Proteins in supernatants were quantified by enzyme-linked immunosorbent assay (ELISA) using a corresponding ELISA kit (R&D Systems, Minneapolis, MN, USA), according to the manufacturer’s instructions.

### 2.7. Detection of Gene Expression in TNF-α-Treated HDF

Experiments were conducted as previously described [28]. Briefly, HDFs were plated at a density of 3 × 10^5^ cells/well in 6-well plates, allowed to adhere overnight, and then starved for 24 h under serum-free conditions. HDFs were challenged with 20 ng/mL TNF-α in the presence or absence of MTF (12.5 and 25 µg/mL), AIF (25 and 50 µM), and MAIF (25 and 50 µM) for 4 h (for IL-1β, IL-6, and IL-8), and 24 h (for MMP-1 and COLIA1). Cells’ mRNA expression was quantified by quantitative real-time polymerase chain reaction (qRT-PCR). Cells’ RNA was extracted using the RNeasy Mini Kit (Qiagen, Germantown, MD, USA) according to the manufacturer’s instructions. The cDNA was synthesized from RNA using the RevertAid First Strand cDNA Synthesis kit (Thermo Fisher Scientific, Waltham, MA, USA). The PCR reaction was conducted with PowerUp SYBR PCR Master Mix (Applied Biosystems, Waltham, MA, USA), each corresponding primer, and synthesized cDNA using the following thermal conditions: initial denaturation at 95 °C for 10 min, followed by 40 cycles with denaturation at 95 °C for 1 s, annealing and elongation at 60 °C for 30 s. The mRNA expression was normalized to that of the β-actin reference gene. The primers used are shown in Table 1. The analysis was performed using the Quant Studio 3 real-time PCR system (Applied Biosystems). The relative gene expression was calculated compared to an untreated group using the comparative threshold C_t_ method.

### 2.8. Detection of Protein Expression in TNF-α-Treated HDF

Experiments were conducted as previously described [29]. Briefly, HDFs were plated at a density of 3 × 10^5^ cells/well in 6-well plates, allowed to adhere overnight, and then starved for 24 h under serum-free conditions. HDFs were challenged with 20 ng/mL TNF-α in the presence or absence of MTF (12.5 and 25 µg/mL), AIF (25 and 50 µM), and MAIF (25 and 50 µM) for 15 min (for phospho-extracellular signal-regulated kinase (p-ERK), ERK, phospho-C-Jun, T-terminal kinase (p-JNK), JNK, p-p38, p38, and glyceraldehyde 3-phosphate dehydrogenase (GAPDH)), 4 h (for nuclear factor-kappa B (NF-κB) and activator protein-1 (AP-1)), and 6 h (for iNOS and COX-2). The cells’ protein expression was quantified by Western blot analysis. Cell lysates were extracted using radioimmunoprecipitation assay buffer (RIPA buffer; Tech & Innovation, Gangwon, Korea). Protein concentration was determined using the Pierce™ BCA Protein Assay Kit (Pierce, Rockford, IL, USA). Proteins were separated using sodium dodecyl sulfate-polyacrylamide gel electrophoresis (SDS-PAGE). Thereafter, proteins were transferred to a polyvinylidene difluoride membrane (Merck Millipore, Darmstadt, Germany) and blocked with 5% skim milk. The membrane was incubated at room temperature with diluted primary antibodies, including iNOS, COX-2, NF-κB (p65), AP-1, ERK1/2, phospho-ERK1/2, p38, phospho-p38, JNK, phospho-JNK, and GAPDH (Cell Signaling Technology, Danvers, MA, USA), for 4 h. The membrane was washed and incubated at room temperature with rabbit IgG secondary antibody for 1 h. The protein signal was measured using SuperSignal^®^ West Femto Maximum Sensitivity Chemiluminescent Substrate (Pierce) and the Fusion Solo Chemiluminescence System (PEQLAB Biotechnologie GmbH, Erlangen, Germany). The protein expression was normalized to that of GAPDH reference protein. The analysis was performed using a Fusion Solo Chemiluminescence System (PEQLAB Biotechnologie GmbH, Erlangen, Germany). The relative protein expression was calculated compared to an untreated group using the ImageJ software (National Institutes of Health, Bethesda, MD, USA).

### 2.9. Statistical Analyses

All data are expressed as the mean ± standard error of the mean. Statistical analyses were conducted using one-way analysis of variance (ANOVA) and Tukey’s post-test to evaluate differences among the experimental groups (GraphPad Software, Inc., San Diego, CA, USA). *p* < 0.05, *p* < 0.01, and *p* < 0.001 were considered significant.

## 3. Results and Discussion

### 3.1. Intracellular ROS and Pro-Inflammatory Mediator NO Scavenging by MTF, AIF, and MAIF in TNF-α-Treated HDFs

**Figure 1:**
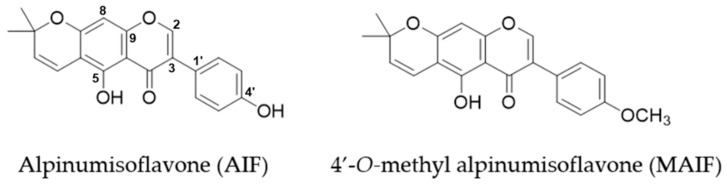
Structure of alpinumisoflavone and 4′-*O*-methyl alpinumisoflavone.

**Figure 2:**
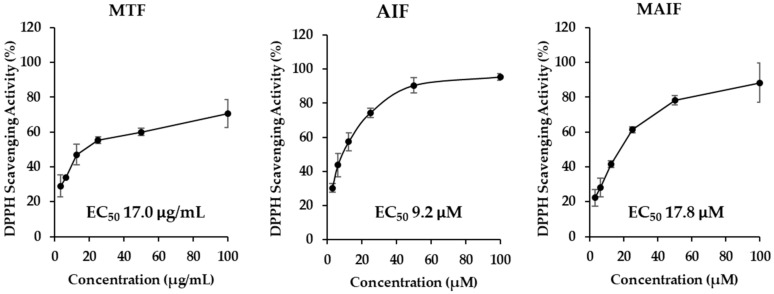
DPPH (2,2-diphenyl-1-picrylhydrazyl) radical scavenging activity by *M. tricuspidata* fruit extract (MTF), alpinumisoflavone (AIF), and 4′-*O*-methyl alpinumisoflavone (MAIF). Each sample was added to DPPH in 96-well plate and mixed. After incubation for 1 h in the dark, the absorbance was measured at 520 nm wavelength using a microplate reader. (EC_50_: Half maximal effective concentration).

In preliminary experiments, *M. tricuspidata* fruit extract (MTF), alpinumisoflavone (AIF), and 4′-*O*-methyl alpinumisoflavone (MAIF) (Figure 1) displayed 1,1-diphenyl-2-picrylhydrazyl (DPPH) radical scavenging activities (Figure 2). The concentration of compound that produces 50% biological effect (EC_50_) of MTF was 17.0 µg/mL. The EC_50_ of AIF and MAIF were at 9.2 and 17.8 μM, respectively. Previous studies have reported that various isoflavones acted as antioxidants and inhibited oxidative stress within cells [30,31,32,33]. Therefore, we focused our research on the possibility that these isoflavones will inhibit oxidative stress in HDFs.

As described above, exposure to UV radiation increases the accumulation of intercellular ROS and the secretion of pro-inflammatory cytokines such as TNF-α. Additionally, ROS produced by mitochondria serve as signaling molecules that upregulate inflammatory cytokines including TNF-α. Excessively elevated TNF and ROS regulate and activate the elevated levels of each other that lead to collagen cleavage and various inflammatory responses. Therefore, ROS and TNF-α can be used as substitute inducers because they act by a mechanism similar to skin aging and inflammatory response resulting from UV exposure. We used TNF-α as a marker to evaluate the inhibitory effects of MTF, AIF, and MAIF on ROS accumulation and NO production.

**Figure 3:**
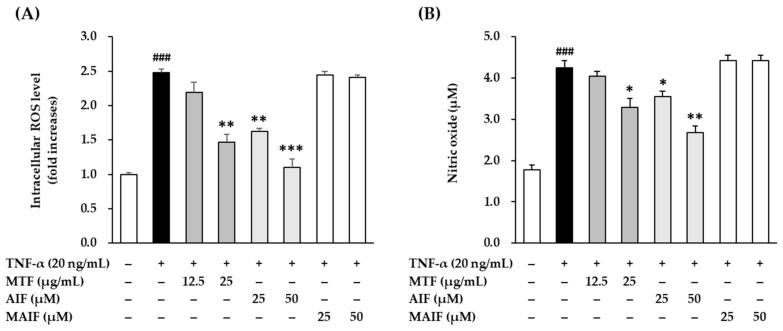
Scavenging of intracellular reactive oxygen species (ROS) (**A**) and pro-inflammatory mediator nitric oxide (NO) (**B**) by *M. tricuspidata* fruit extract (MTF), alpinumisoflavone (AIF), and 4′-*O*-methyl alpinumisoflavone (MAIF) in tumor necrosis factor-α (TNF-α)-treated human dermal fibroblasts (HDFs). HDFs were challenged with 20 ng/mL TNF-α in the presence or absence of MTF (12.5 and 25 µg/mL), AIF (25 and 50 µM), and MAIF (25 and 50 µM) for 30 min or 24 h. The levels of ROS and NO were quantified by using 2′, 7′-dichlorofluorescin diacetate (DCFDA) stain and Griess assay. The data are presented as mean ± SEM of at least three independent experiments. ^###^ *p* < 0.001 as compared with the untreated cells. * *p* < 0.05, ** *p* < 0.01 and *** *p* < 0.01 as compared with the TNF-α-treated cells.

To investigate the antioxidant potential of MTF, AIF, and MAIF, the inhibition of intracellular ROS production after TNF-α treatment in HDFs was quantified with DCFDA fluorogenic dye. Because the extract and two isoflavones were not cytotoxic to HDFs at 25 μg/mL and 50 μM, respectively (data not shown), experiments were performed using these concentrations. Intracellular ROS production after 30 min of adding 20 ng/mL TNF-α revealed that the basal ROS production in HDFs was 2.48 ± 0.06-fold (*p* < 0.001) compared to the untreated cells (Figure 3A). The production was inhibited to 2.19 ± 0.16-fold (not significant) and 1.47 ± 0.11-fold (*p* < 0.05) by MTF at 12.5 and 25 µg/mL concentrations, respectively. Treatment with AIF alone suppressed the intracellular ROS production in TNF-α-treated HDFs to significantly lower levels (25 µM: 1.63 ± 0.04-fold, *p* < 0.01; 50 µM: 1.10 ± 0.12-fold, *p* < 0.001), whereas MAIF did not. These results showed that MTF extract and compound AIF inhibited ROS accumulation in TNF-α-treated HDFs, indicating a possibility that these two agents might attenuate the skin damage caused by oxidative stress.

To assess the anti-inflammatory potential, the inhibition of pro-inflammatory NO production in HDFs after TNF-α treatment was quantified by the Griess assay. NO production after 24 h of 20 ng/mL TNF-α administration revealed that the NO secretion in HDF was 4.24 ± 0.18 µM (2.39-fold, *p* < 0.001) compared to the untreated cells (1.78 ± 0.12µM, 1-fold) (Figure 3B). Similar to the ROS results described above, AIF suppressed significantly NO secretion in TNF-α-treated HDFs (25 µM: 3.55 ± 0.14 µM, 2.00-fold, *p* < 0.05; 50 µM: 2.69 ± 0.15 µM, 1.51-fold, *p* < 0.01), whereas MAIF did not lower NO secretion. These results indicated that NO production was inhibited in TNF-α-treated HDFs by compound AIF that may inhibit skin damage caused by the inflammatory response.

Taken together, these results indicate that AIF removes excess ROS and suppresses NO production but MAIF does not. AIF and MAIF presented a typical pyranoisoflavone structure, which is a type of isoflavone. In moieties of isoflavone, they equally attached with the one a pyran group to carbon positions 6 and 7 (C-6, 7). They substituted the hydroxyl group or the methoxy group at C-4′. In detail, AIF was attached with the hydroxyl groups, in contrast, MAIF was attached with the methoxy group. Therefore, the potential of AIF against ROS scavenging and NO inhibition was considered in correlation with hydroxyl group substitution of C-4′. Base on these results, we focused on AIF in the subsequent experiments.

### 3.2. Inhibition of COX-2 and iNOS Expression by AIF in TNF-α-Treated HDFs

Because the activation of COX-2 and iNOS plays an important role in NO production, we evaluated the effect of AIF on COX-2 and iNOS protein expression increased by TNF-α stimulation in HDFs.

**Figure 4:**
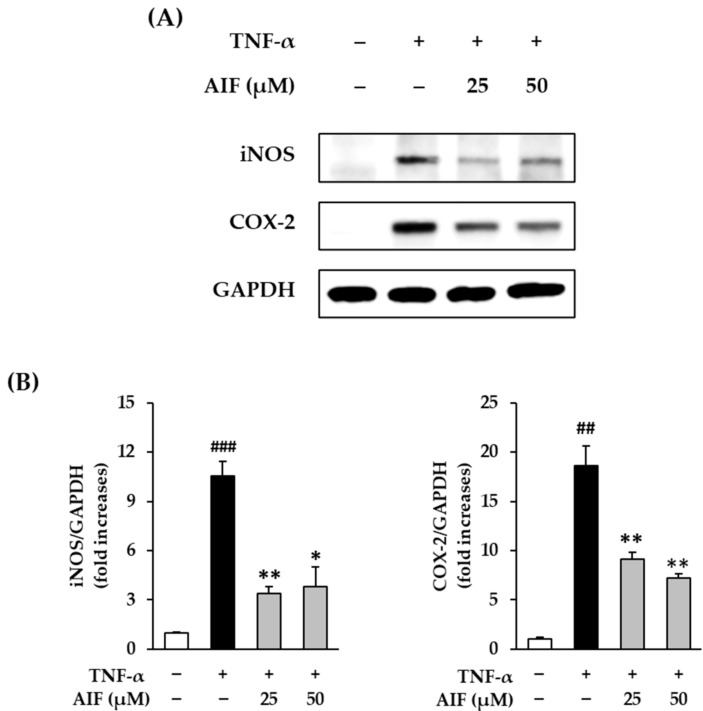
Inhibition of cyclooxygenase-2 (COX-2) and inducible nitric oxide synthase (iNOS) protein expression by alpinumisoflavone (AIF) in tumor necrosis factor-α (TNF-α)-treated human dermal fibroblasts (HDFs). (**A**) Expression of COX-2, iNOS, and glyceraldehyde 3-phosphate dehydrogenase (GAPDH) proteins. (**B**) Relative protein expression levels of COX-2 and iNOS. HDFs were challenged with 20 ng/mL TNF-α in the presence or absence of AIF (25 and 50 µM) for 6 h. The protein expression was determined using Western blot analysis. The data are presented as mean ± SEM of at least three independent experiments. ^##^ *p* < 0.01 and ^###^ *p* < 0.001 as compared with the untreated cells. * *p* < 0.05 and ** *p* < 0.01 as compared with the TNF-α-treated cells.

The expression of COX-2 and iNOS proteins in TNF-α-treated HDFs in the presence of AIF is shown in Figure 4. The protein expression of COX-2 after 6 h of 20 ng/mL TNF-α administration was 10.6 ± 0.85-fold (*p* < 0.01) compared to the untreated cells (Figure 4A). AIF administration in TNF-α-treated HDFs evidently increased the expression of COX-2 (25 µM: 3.38 ± 0.40-fold, *p* < 0.05; 50 µM: 3.80 ± 1.19-fold, *p* < 0.05) but the change was not concentration-dependent (Figure 4B). TNF-α treatment significantly increased the expression of iNOS (18.6 ± 2.08-fold, *p* < 0.01) compared to the untreated group (Figure 4A). iNOS expression was significantly inhibited to 9.13 ± 0.65 (*p* < 0.01) and 7.19 ± 0.48-fold (*p* < 0.01) by 25 and 50 µM of AIF, respectively (Figure 4B). These results suggest that AIF may suppress inflammation in TNF-α-treated HDFs.

Previous studies reported that various isoflavones suppress NO and COX-2 and inhibit the inflammatory response via the NF-κB pathway [34,35]. Consistent with previous reports, AIF inhibited the inflammatory response of iNOS and COX-2 in TNF-α-treated HDFs. Therefore, it is proposed that AIF can reduce inflammation induced by the accumulation of ROS.

### 3.3. Inhibition of MMP-1 and COLIA1 Expression by AIF in TNF-α-Treated HDFs

The ECM of the skin is composed of a complex of collagen and non-collagen components. External stimuli, including UV radiation, leads to the accumulation of ROS that alters the structure of genes and proteins, including collagen and collagen-degrading enzymes. Ultimately, such changes damage the skin ECM and induce aging-related phenomena [36,37]. Because MMP-1 is a collagenase that plays a key role in skin collagen degradation, inhibitors of MMP-1 may be potential candidates for preventing skin aging associated with wrinkle formation and sagging [38]. Therefore, we evaluated MMP-1 expression in TNF-α-treated HDFs.

**Figure 5:**
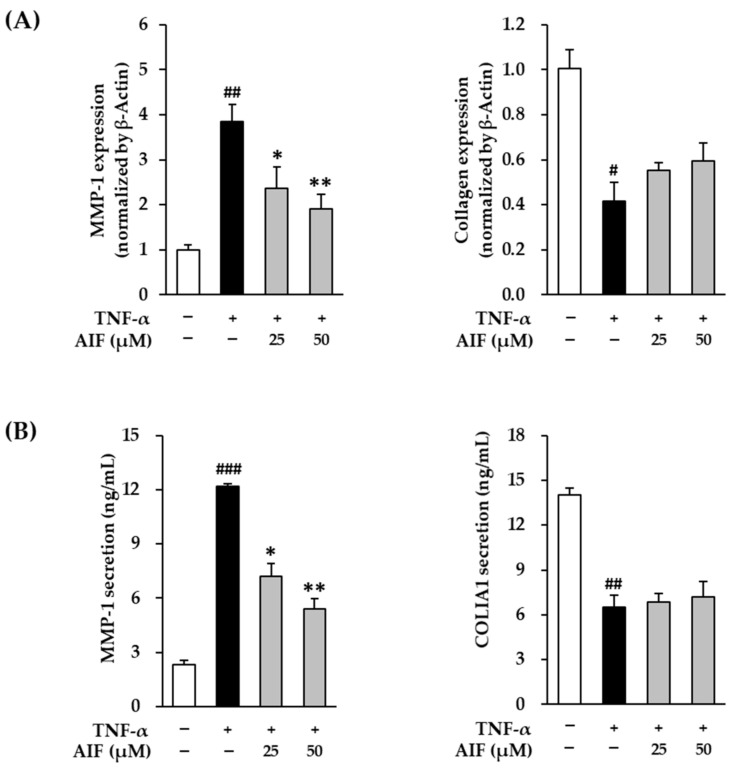
Effect of alpinumisoflavone (AIF) on matrix metalloproteinase-1 (MMP-1) and type I collagen (COLIA1) expression in tumor necrosis factor-α (TNF-α)-treated human dermal fibroblasts (HDFs). (**A**) Relative mRNA expression levels of *MMP-1* and *COLIA1.* (**B**) Secretion of MMP-1 and COLIA1 proteins. HDFs were challenged with 20 ng/mL TNF-α in the presence or absence of AIF (25 and 50 µM) for 4 or 12 h. The protein expression was determined using qRT-PCR and ELISA. The data are presented as mean ± SEM of at least three independent experiments. ^#^ *p* < 0.05, ^##^ *p* < 0.01 and ^###^ *p* < 0.001 as compared with the untreated cells. * *p* < 0.05 and ** *p* < 0.01 as compared with the TNF-α-treated cells.

The mRNA expression and protein secretion of MMP-1 collagenase in TNF-α-treated HDFs treated with AIF are shown in Figure 5. The mRNA expression of *MMP-1* collagenase in HDFs after 4 h of 20 ng/mL TNF-α administration was 3.86 ± 0.37-fold (*p* < 0.01) compared with the untreated cells (Figure 5A). AIF administration significantly increased the expression of MMP-1 (25 µM: 2.36 ± 0.48-fold, *p* < 0.05; 50 µM: 1.92 ± 0.32-fold, *p* < 0.01) in a concentration-dependent manner. Analogously, after TNF-α treatment, protein secretion of MMP-1 (12.2 ± 0.17 ng/mL, *p* < 0.01) was significantly increased compared with the untreated group (2.31 ± 0.22 ng/mL), whereas it was significantly suppressed to 7.21 ± 0.72 ng/mL (*p* < 0.05) and 5.43 ± 0.53 ng/mL (*p* < 0.01) by 25 and 50 µM of AIF, respectively (Figure 5B).

Procollagen is a precursor molecule containing additional peptide sequences in collagen synthesis. Some sequences can provide indirect information on collagen synthesis because these sequences are cleaved during collagen secretion. Therefore, procollagen COLIA1 expression was measured to assess collagen synthesis levels.

TNF-α administration inhibited the mRNA expression of COLIA1 after 4 h of 20 ng/mL 0.42 ± 0.08-fold (*p* < 0.05) compared to the untreated cells (Figure 5A). AIF administration in TNF-α-treated HDFs tended to increase the expression of COLIA1 (25 µM: 0.55 ± 0.04-fold; 50 µM: 0.59 ± 0.08-fold). Similarly to mRNA results, protein secretion of COLIA1 (6.49 ± 0.82 ng/mL, *p* < 0.01) was significantly increased after TNF-α treatment compared with the untreated group (14.01 ± 0.46 ng/mL) (Figure 5A). However, it was not affected by treatment with 25 and 50 µM of AIF (Figure 5B). These results indicate that although AIF recovered the gene expression, it did not change significantly procollagen expression in TNF-α-stimulated HDFs. Regardless, AIF might potentially suppress enhanced skin ECM degradation caused by oxidative stress.

### 3.4. Inhibition of Pro-Inflammatory Cytokines IL-1β, IL-6, and IL-8 by AIF in TNF-α-Treated HDFs

Pro-inflammatory cytokines, such as TNF-α, IL-1β, IL-6, and IL-8, are sensitized by cellular oxidative stress and upregulate the inflammatory response [39,40]. These inflammatory reactions induce aging and various lesions in the skin [12,41]. To evaluate whether AIF suppresses the inflammatory response in skin cells, IL-1β, IL-6, and IL-8 mRNA gene expression was directly measured in TNF-α-treated HDFs.

**Figure 6:**
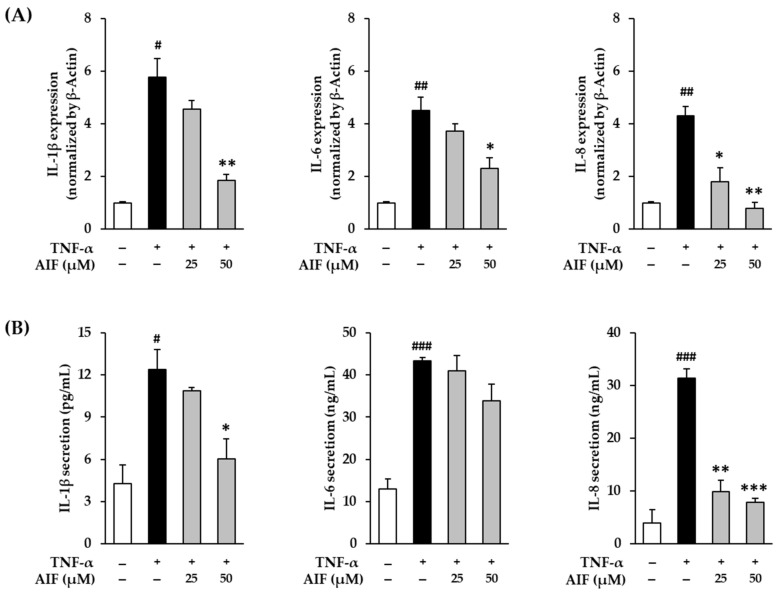
Inhibition of interleuk (IL)-1β, IL-6, and IL-8 expression by alpinumisoflavone (AIF) in tumor necrosis factor-α (TNF-α)-treated human dermal fibroblasts (HDFs). (**A**) Relative mRNA expression levels of *IL-1β, IL-6, and IL-8.* (**B**) Secretion of IL-1β, IL-6, and IL-8. HDFs were challenged with 20 ng/mL TNF-α in the presence or absence of AIF (25 and 50 µM) for 4 or 12 h. The protein expression was determined using qRT-PCR and ELISA. The data are presented as mean ± SEM of at least three independent experiments. ^#^ *p* < 0.05, ^##^ *p* < 0.01 and ^###^ *p* < 0.001 as compared to untreated cells. * *p* < 0.05, ** *p* < 0.01 and *** *p* < 0.001 as compared to TNF-α-treated cells.

The mRNA expression and secretion of IL-1β, IL-6, and IL-8 proteins in TNF-α-treated HDFs treated with AIF are shown in Figure 6. The mRNA expression of *IL-1β*, *IL-6*, and *IL-8* after 4 h of 20 ng/mL TNF-α administration increased 5.78 ± 0.72-fold (*p* < 0.05), 4.50 ± 0.53-fold (*p* < 0.01), and 4.32 ± 0.36-fold (*p* < 0.01), respectively, compared to untreated cells (Figure 6A). Compared to the TNF-α-treated group, AIF administration decreased the expression of *IL-1β* (25 µM: 4.56 ± 0.33-fold; 50 µM: 1.86 ± 0.21-fold, *p* < 0.01), *IL-6* (25 µM: 3.72 ± 0.28-fold; 50 µM: 2.30 ± 0.42-fold, *p* < 0.01), and *IL-8* (25 µM: 1.80 ± 0.54-fold; 50 µM: 0.78 ± 0.25-fold, *p* < 0.01).

To evaluate the change in the actual secretion of pro-inflammatory cytokines, ELISA was performed. Analogously with mRNA results, secretion of IL-1β, IL-6, and IL-8 proteins in HDFs after 12 h of 20 ng/mL TNF-α administration increased to 12.4 ± 1.46 ng/mL (*p* < 0.05), 43.4 ± 0.84 ng/mL (*p* < 0.001), and 31.4 ± 1.89 ng/mL (*p* < 0.001) compared to untreated cells (IL-1β: 4.28 ± 1.33 ng/mL, IL-6: 12.9 ± 2.42 ng/mL, IL-8 3.98 ± 2.53 ng/mL, respectively (Figure 6B). The secretion of IL-8 was significantly suppressed to 9.87 ± 2.11-fold (*p* < 0.01) and 7.87 ± 0.74-fold (*p* < 0.001) by 25 and 50 µM of AIF, respectively. The secretion of IL-8 was also reduced by AIF, however, only at 50 µM (6.05 ± 1.42-fold, *p* < 0.05) concentration. These results indicate that AIF may suppress skin inflammatory responses against TNF-α stimulation by inhibiting pro-inflammatory cytokines. Therefore, AIF may attenuate inflammation-related skin aging and diseases.

### 3.5. Inhibition of NF-κB and AP-1 Expression by AIF in TNF-α-Treated HDFs

MMP-1 and pro-inflammatory cytokines are downregulated by suppressing AP-1 and NF-κB activation. Therefore, AIF was expected to suppress MMP-1 and pro-inflammatory cytokine levels and promote collagen synthesis. To evaluate the role of the AP-1 and NF-κB pathways in the action of AIF, Western blot analysis and immunofluorescence staining were performed.

**Figure 7:**
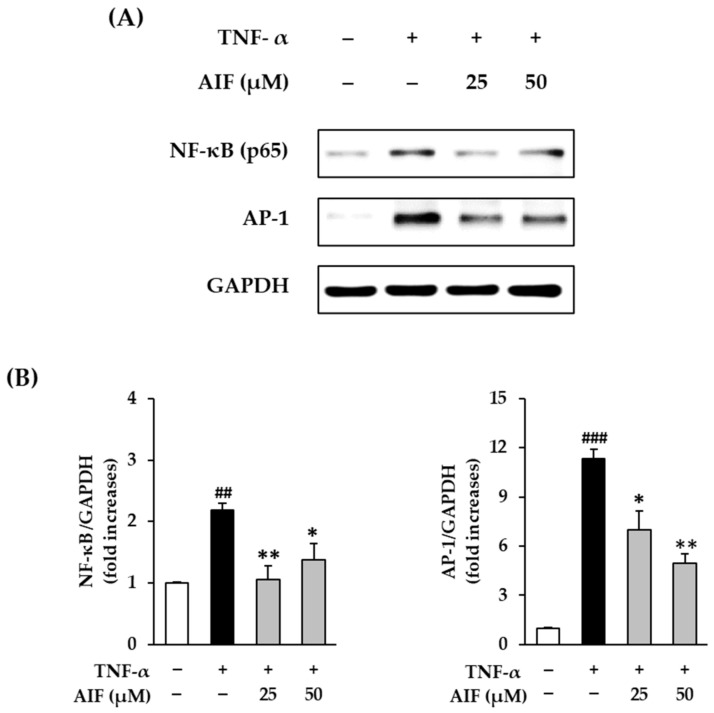
Inhibition of nuclear factor-κB (NF-κB) (p65) and activator protein 1 (AP-1) protein expression by alpinumisoflavone (AIF) in tumor necrosis factor-α (TNF-α)-treated human dermal fibroblasts (HDFs). (**A**) Protein expression of NF-κB, AP-1, and glyceraldehyde 3-phosphate dehydrogenase (GAPDH). (**B**) Relative protein expression levels of NF-κB (p65) and AP-1. HDFs were challenged with 20 ng/mL TNF-α in the presence or absence of AIF (25 and 50 µM) for 12 h. The protein expression was determined using Western blot analysis. The data are presented as mean ± SEM of at least three independent experiments. ^##^ *p* < 0.01 and ^###^ *p* < 0.001 as compared to untreated cells. * *p* < 0.05 and ** *p* < 0.01 as compared to TNF-α-treated cells.

NF-κB (p65) and AP-1 protein expression after 12 h of 20 ng/mL TNF-α administration increased compared to untreated cells (Figure 7A). As shown in Figure 7B, the relative protein expression of NF-κB (p65) and AP-1 was significantly increased to 2.18 ± 0.12-fold (*p* < 0.01) and 11.3 ± 0.60-fold (*p* < 0.001) by 20 ng/mL TNF-α administration, respectively. The protein expression of NF-κB (p65) was dramatically inhibited by AIF; however, the inhibition was not concentration-dependent (25 µM: 1.06 ± 0.22-fold, *p* < 0.01; 1.37 ± 0.27-fold, *p* < 0.05). Expression of AP-1 was suppressed by AIF in a concentration-dependent manner (25 µM: 6.97 ± 1.17-fold, *p* < 0.05; 4.97 ± 0.55-fold, *p* < 0.01).

**Figure 8:**
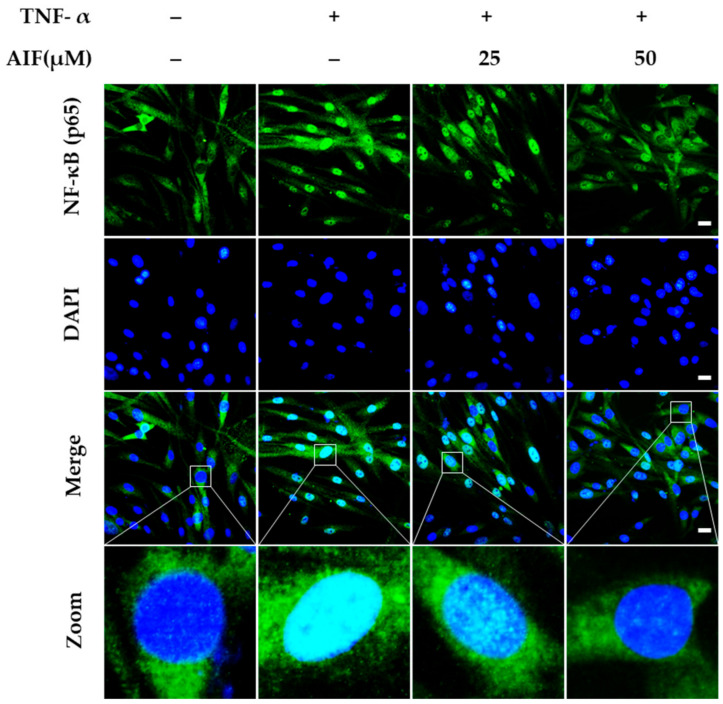
Inhibitory effect of nuclear factor-κB (NF-κB) (p65) translocation by alpinumisoflavone (AIF) in tumor necrosis factor-α (TNF-α)-treated human dermal fibroblasts (HDFs). AIF in TNF-α-treated HDFs. HDFs were challenged with 20 ng/mL TNF-α in the presence or absence of AIF (25 and 50 µM) for 3 min. The expression of NF-κB (p65) was visualized using immunofluorescence staining (40× magnification, scale bar: 20 μm).

In Figure 8, NF-KB(p65) was located in cytoplasm in untreated cells, however it was translocated into the nucleus by TNF-α treatment. The TNF-α-induced nuclear translocation of NF-icBp65 was supressed by AIF treatment. These results indicate that AIF may suppress skin inflammatory responses against TNF-α stimulation by downregulating AP-1 and NF-κB activation. Therefore, AIF may have the potential to help enhance inflammation-related skin aging and diseases.

### 3.6. Inhibition of MAPK Phosphorylation by AIF in TNF-α-Treated HDFs

The AP-1 and NF-κB pathways regulate MMP-1 and pro-inflammatory cytokines, and these pathways are upregulated by MAPK activation [42]. To assess whether AIF can suppress MAPK phosphorylation by TNF-α stimulation, we evaluated the action of AIF using Western blot analysis.

**Figure 9:**
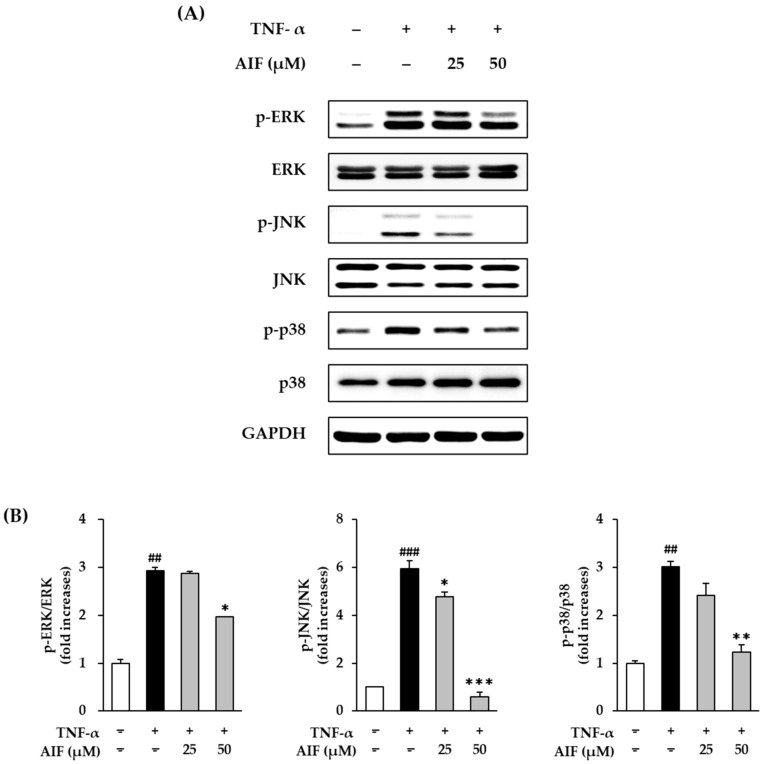
Inhibition of mitogen-activated protein kinase (MAPK) phosphorylation by alpinumisoflavone (AIF) in tumor necrosis factor-α (TNF-α)-treated human dermal fibroblasts (HDFs). (**A**) Expression of p-extracellular signal-regulated kinase (ERK), ERK, p- c-Jun N-terminal kinases (JNK), JNK, p-p38, p38, and glyceraldehyde 3-phosphate dehydrogenase (GAPDH) proteins. (**B**) Relative expression levels of p-ERK/ERK, p-JNK/JNK, p-p38/p38 proteins. HDFs were challenged with 20 ng/mL TNF-α in the presence or absence of AIF (25 and 50 µM) for 15 min. The protein expression was determined using Western blot analysis. The data are presented as mean ± SEM of at least three independent experiments. ^##^ *p* < 0.01 and ^###^ *p* < 0.001 as compared to untreated cells. * *p* < 0.05, ** *p* < 0.01 and *** *p* < 0.001 as compared to TNF-α-treated cells.

The MAPK protein expression after 15 min of 20 ng/mL TNF-α administration increased, compared to that in untreated cells (Figure 9A). Treatment with AIF suppressed MAPKs phosphorylation. As shown in Figure 9B, phosphorylation was significantly augmented by 2.93 ± 0.07-fold for MAPK, for ERK, *p* < 0.01, 5.94 ± 0.34-fold (JNK, *p* < 0.001), and 3.02 ± 0.12-fold (p38, *p* < 0.01) by 20 ng/mL TNF-α administration, respectively. The phosphorylation of ERK was decreased in the AIF-treated group to 2.87 ± 0.07-fold (25 µM, no significant) and 1.97 ± 0.01-fold (50 µM, *p* < 0.05). The phosphorylation of JNK was significantly inhibited in the AIF-treated group to 4.76 ± 0.21-fold (25 µM, *p* < 0.05) and 0.60 ± 0.19-fold (50 µM, *p* < 0.001). The phosphorylation of p38 was reduced in the AIF-treated group to 2.41 ± 0.25-fold (25 µM, not significant) and 1.07 ± 0.16-fold (50 µM, *p* < 0.01). These results indicate that AIF may suppress AP-1 and NF-κB activation induced by TNF-α by suppressing the phosphorylation of MAPK.

UV light directly or indirectly induces ROS and pro-inflammatory mediators, such as NO, iNOS, COX-2, IL-1β, IL-6, and IL-8 [15,16,17]. These molecules are associated with skin damage and sustained accelerated photoaging by UV exposure [15,18]. Many antioxidants have been reported to prevent photoaging of the skin by suppressing ROS accumulation and downregulation of AP-1, NF-κB, and MAPKs [43,44,45]. MAPK activation induces excessive synthesis of collagenase MMP-1 and degrades ECM [46]. ROS induced by TNF-α stimulation also induces degradation of skin ECM due to activation of AP-1, NF-κB, and MAPKs [12,41]. Therefore, the suppression of AP-1, NF-κB, and activation of MAPK activation by AIF demonstrates that the compound inhibits the inflammatory response and MMP-1 synthesis.

Taken together, AIF isolated from MTF has antioxidant and inflammatory effects through the inhibition of ROS and NO accumulation in TNF-α-induced HDFs. AIF can inhibit the degradation of skin ECM by increasing MMP-1 collagenase and decreasing collagen synthesis. AIF has been associated with the inhibition of iNOS and activation of COX-2 and pro-inflammatory cytokines IL-1β, IL-6, and IL-8. Inhibition of TNF-α-induced activation of NF-κB, AP-1, and MAPKs in HDFs by AIF suggests its possible utility in attenuating skin aging.

## 4. Conclusions

ROS is a major part of the inflammatory response and ECM degradation that leads to skin aging and various cutaneous lesions. Therefore, ROS inhibitors can be used to attenuate skin aging and disease. This study shows that alpinumisoflavone (AIF) isolated from *M. tricuspidata fruit* inhibits TNF-α-induced ROS, NO, and MMP-1 expression and increased collagen synthesis. AIF reduces the expression of TNF-α-induced pro-inflammatory cytokine mediators, including iNOS and COX-2, and the pro-inflammatory cytokines IL-1β, IL-6, and IL-8. The mechanism by which AIF inhibits TNF-α-induced responses in HDF is mediated through inhibition of NF-κB, AP-1, and MAPK activation. Our findings provide the first evidence that AIF may be effective in attenuating skin damage caused by oxidative stress. Although further research is needed fully to understand the mechanisms of AIF’s activity, the compound is a potential agent for inhibiting skin aging and various cutaneous lesions.

## Figures and Tables

**Table 1:** List of primer sequences.

Genes	Sequences	
Matrix metalloproteinase-1 (AF158733)	Sense	5′-ATTCTACTGATATCGGGGCTTT-3′
	Antisense	5′-ATGTCCTTGGGGTATCCGTGTA-3′
Procollagen I α1 (X07884)	Sense	5′-CTCGAGGTGGACACCACCCT-3′
	Antisense	5′-CAGCTGGATGGCCACATCGG-3′
Interleukin-1β (NM_000576)	Sense	5′-CTGTCCTGCGTGTTGAAAGA-3′
	Antisense	5′-TTCTGCTTGAGAGGTGCTGA-3′
Interleukin-6 (HUMIL6CSF)	Sense	5′-CAGGAATTGAATGGGTTTGC-3′
	Antisense	5′-AAACCAAGGCACAGTGGAAC-3′
Interleukin-8 (HUMIL8A)	Sense	5′-CTCCTTCTCCACAAGCGCC-3′
	Antisense	5′-GCCGAAGAGCCCTCAGGC-3′
β-Actin (DQ407611)	Sense	5′-AGAGATGGCCACGGCTGCTT-3′
	Antisense	5′-ATTTGCGGTGGACGATGGAG-3′
